# Personalised immunotherapy strategies informed by single cell profiling in thyroid cancer: a mini review

**DOI:** 10.3389/fimmu.2025.1651088

**Published:** 2025-09-01

**Authors:** Ruyu Chen, Zhimin Wang, Xueying Chen, Yangling Huang, Fengnuan Zhang, Tianshu Gao

**Affiliations:** ^1^ First Clinical College, Liaoning University of Traditional Chinese Medicine, Shenyang, Liaoning, China; ^2^ Department of Endocrinology,The First Affiliated Hospital of Liaoning University of Traditional Chinese Medicine, Shenyang, Liaoning, China

**Keywords:** single-cell RNA-sequencing, spatial transcriptomics, tumour microenvironment, thyroid cancer, immune checkpoint blockade, macrophage reprogramming

## Abstract

Thyroid cancer (TC) is now among the fastest-growing solid tumours, yet therapeutic gains remain limited for poorly differentiated, anaplastic and medullary variants whose median survivals are measured in months. Once guided chiefly by histology and single-gene assays, immunotherapy is being reshaped by single-cell profiling, which exposes the cellular mosaics that arbitrate response and resistance. Dissection of more than 150–000 tumour- and immune-cell transcriptomes has uncovered follicular-like, partial EMT-like and dedifferentiated thyrocyte states embedded within ‘hot’ (CD8^hi^ IFN-γ^hi^), ‘cold’ (CD8^lo^) and ‘excluded’ (stroma-walled) immune niches; these phenotypes correlate with PD-1/LAG-3 expression, macrophage polarisation and radio-iodine refractoriness. Functional studies reveal that SPP1–CD44 and GAS6–AXL crosstalk licenses epithelial–mesenchymal transition while VSIG4^+^ macrophages blunt cytotoxic T-cell activity, collectively undermining checkpoint blockade. Spatial transcriptomics corroborates these insights, mapping PD-L1-high tumour islets millimetres from CXCL13-rich tertiary lymphoid structures, whereas CITE-seq quantifies actionable checkpoints and cytokine receptors across patient biopsies. Emerging therapeutics mirror this granular knowledge: combinatorial PD-1 + LAG-3 inhibition, CSF-1R-directed macrophage re-programming and TSH-receptor-targeted CAR-T cells are advancing through early-phase trials, while ex-vivo single-cell pharmacotyping aligns drug cocktails with an individual’s tumour ecosystem. Early lenvatinib-pembrolizumab or selpercatinib-nivolumab trials show ~40% ORR but grade-3 hypertension >60%, prompting staggered-start designs. These advances sharpen pathogenetic resolution, refine patient selection and accelerate translational pipeline design. By integrating single-cell biology, immunology and endocrine oncology, this review identifies diagnostic blind spots, spotlights drug-repurposing opportunities and charts a roadmap toward personalised immunotherapeutic strategies capable of improving outcomes across the diverse spectrum of thyroid cancer.

## 1 Introduction

Thyroid cancer (TC) now ranks among the fastest-rising solid tumours worldwide, accounting for ≈ 3% of all new cancers and > 821–000 incident cases in 2022 alone (1, 2). While age-standardised mortality has fallen over the past three decades, the absolute burden continues to climb—Global Burden of Disease 2021 data record > 249–000 cases and > 44–000 deaths in 2021, with an annual incidence increase of 1.25% since 1990 (3, 4). Most differentiated tumours respond to surgery, radioactive iodine, and TSH suppression, yet up to 15% recur, and poorly differentiated, anaplastic, and medullary subtypes retain dismal survival, underscoring an unmet therapeutic need.

Immune-checkpoint blockade has begun to address that gap, pembrolizumab received FDA approval for advanced TC in 2020, and > 80 clinical trials launched since 2018 explore PD-1/PD-L1, CTLA-4, bispecific antibodies, cytokine therapy, and adoptive platforms such as CAR-T and TCR-T (5, 6). Nevertheless, objective responses remain inconsistent and immune-related endocrine toxicities—most notably thyroiditis and hypothyroidism—complicate prolonged treatment (7, 8). These observations suggest that bulk histology and single-gene biomarkers are insufficient to predict benefit or guide escalation.

Single-cell profiling is transforming this landscape by deconvoluting the cellular and molecular heterogeneity that governs therapeutic sensitivity. A 158 577-cell atlas of papillary thyroid carcinoma (PTC) spanning local disease to radio-iodine–refractory metastases delineated three malignant thyrocyte phenotypes (follicular-like, partial EMT-like, dedifferentiation-like) and uncovered a BRAF-like-B subtype enriched for cancer-associated fibroblasts and immunotherapy-responsive gene programmes (9, 10). Parallel integrative analyses combining bulk and single-cell RNA-seq have mapped eight tumour-cell subclusters and ligand–receptor circuits that stratify prognosis and nominate actionable pathways (11, 12). At the metastatic frontier, single-cell dissection of lymph-node deposits highlights divergent myeloid and T-cell states that correlate with nodal architecture and future relapse risk (13, 14). Collectively, these studies show that seemingly uniform thyroid tumours harbour “hot” (TIL-rich), “cold” (immune-desert) and “excluded” (peripheral-T-cell) niches in close proximity, each demanding tailored intervention.

Beyond mechanistic insight, single-cell techniques furnish practical tools for personalisation: predictive gene signatures, multiplex spatial maps for biopsy triage, and ex vivo drug-response platforms integrating transcriptomic and T-cell–receptor read-outs (15–17). Such resolution is pivotal for matching patients to immune checkpoint inhibitors, rational combinations with kinase or anti-angiogenic agents, and next-generation cellular therapies.

This review synthesises emerging evidence at the intersection of single-cell biology and thyroid-cancer immunotherapy. We survey the technological armamentarium illuminating tumour heterogeneity, characterise the immune ecosystem these datasets reveal, and appraise how these insights are informing bespoke therapeutic strategies while spotlighting the challenges that lie ahead.

## 2 Single-cell profiling technologies illuminating thyroid tumour heterogeneity

The past decade has witnessed an exponential rise in single−cell methods that deconstruct thyroid tumours into their constituent genetic, epigenetic, and spatial parts. Each platform answers a distinct biological question; together they create a multi−layered atlas that is already refining patient stratification and therapeutic design. Droplet−based single−cell RNA−sequencing (scRNA−seq) was the inflexion point. However, enzymatic dissociation can release thyroglobulin-laden debris and oxidative stress that cull plasmacytoid dendritic cells, while dropout, batch-correction artefacts and barcode collision further mask rare cells. The 10x Chromium workflow and colleagues delivers >10 000 transcriptomes per run and has become the routine discovery engine for papillary, anaplastic, medullary, and metastatic lesions (18–20). Using this chemistry, some study built a 158 577−cell atlas across the papillary carcinoma continuum that resolved follicular−like, partial−EMT, and dedifferentiated malignant states, each carrying distinct immunotherapy−response programmes (21, 22). In parallel, integrative pipelines that marry bulk and single−cell datasets linked these phenotypes to ligand–receptor pairs (e.g., MDK/LRP1, GAS6/AXL) and to clinical outcome (23, 24). Lymph−node studies extended the approach to metastatic niches, uncovering divergent myeloid and T−cell polarisation trajectories that foreshadow relapse (25). Together, these experiments established scRNA−seq as the foundational lens through which thyroid−tumour heterogeneity is now viewed.

Transcriptional snapshots alone cannot expose the cis−regulatory logic that drives lineage plasticity. Single−cell assay for transposase−accessible chromatin (scATAC−seq), pioneered by Buenrostro et al., profiles open chromatin and transcription−factor occupancy in thousands of nuclei (26, 27). When applied to poorly differentiated and anaplastic thyroid cancers, scATAC−seq revealed pervasive rewiring of thyrocyte enhancers, including de−novo TEAD and FOS−JUN super−enhancers that coincide with immune−checkpoint up−regulation and resistance to MAPK inhibition—mechanistic clues that were invisible in matched scRNA−seq libraries but critical for combination−therapy design. Multiome links enhancer rewiring of PD-L1 and SIGLEC15 to IFN-γ–independent up-regulation, favouring BET-PROTAC combos.

Multi−omic approaches now read out several molecular layers from the same cell. CITE−seq conjugates antibody bar−codes to capture cell−surface proteins alongside mRNA, providing a direct measure of PD−L1, TIM−3, TIGIT, and CTLA−4 abundance on tumour and immune subsets (28, 29). Chromium “Multiome” and other joint−assay chemistries synchronously index transcriptome and chromatin accessibility, enabling inference of enhancer–promoter coupling inside malignant thyrocytes undergoing iodine−refractory transition. Integrative anchors implemented in Seurat v3/v4 harmonise these data across patients, laboratories, and platforms, exposing conserved malignant archetypes that transcend BRAF, RAS, and TERT mutation strata (30, 31).

Because enzymatic dissociation dissolves spatial context, next−generation spatial transcriptomics re−anchors single−cell identities in intact tissue. Technology overviews by Burgess chart the rise of bar−coded slides (10x Visium), bead−based approaches (Slide−seq), and in−situ sequencing chemistries (32, 33). The first Visium study in papillary thyroid carcinoma showed that follicular−like tumour islets localise to well−differentiated peripheries, whereas partial−EMT clusters nestle along invasive edges enriched for cancer−associated fibroblasts; CXCL13^+^ tertiary lymphoid structures and PD−L1 high tumour foci coexist within millimetres yet display opposing cytokine milieus, validating the “hot−versus−cold” mosaic predicted by dissociated data (34, 35). Visium’s 55 µm spots blur micro-interfaces; we therefore set adaptive filters—typically <15% mitochondrial genes but relaxed to <25% in Hurthle-cell variants—alongside >800-UMI cut-offs when filtering thyroid slides. Similar maps in anaplastic carcinoma have begun to identify peri−necrotic macrophage belts that express SPP1 and VEGFA, nominating spatially confined targets for macrophage−reprogramming strategies.

High−parameter proteomic cytometry completes the picture where RNA degrades or FFPE archives prevail. Mass cytometry (CyTOF), conceptualised by Spitzer and Nolan, tags antibodies with heavy−metal isotopes to quantitate >40 proteins per cell without spectral overlap (36, 37). Applied to fresh thyroidectomy specimens, CyTOF quantifies exhausted PD−1hiTIM−3^+^ CD8^+^ T cells, CCL18^+^CD163^+^ M2−like macrophages, and tissue−resident memory T cells, corroborating transcriptional predictions while delivering pharmacodynamic markers suitable for early−phase immunotherapy trials. Emerging imaging mass−cytometry and multiplex−ion−beam imaging add sub−cellular resolution, delineating checkpoint−enriched immunological synapses at the tumour–stroma interface. Formalin-fixed thyroidectomies create RNA–protein cross-links that impede CITE-seq antibody capture, necessitating FFPE-compatible spatial bar-coding workflows to rescue protein readouts.

## 3 Immune ecosystem of thyroid cancer revealed by single-cell atlases

**Figure 1:**
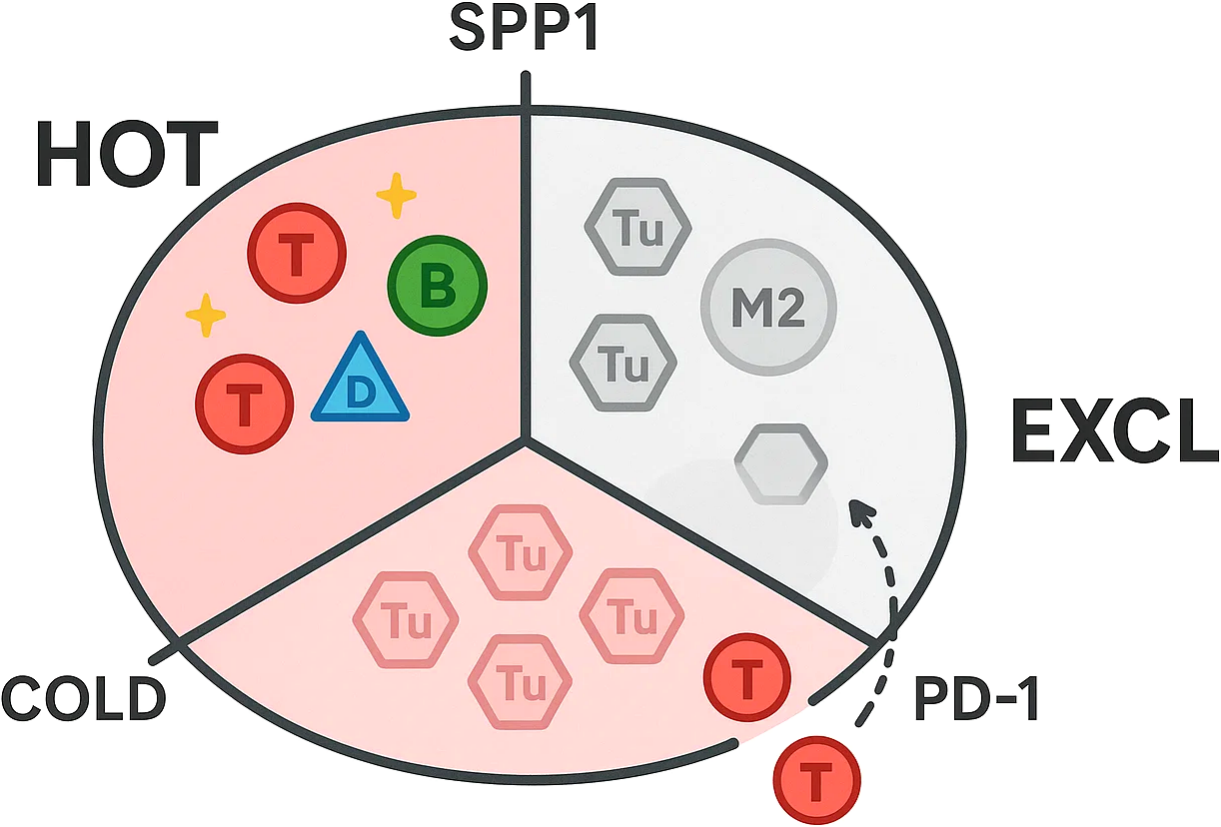
Hot, cold, and excluded immune niches in thyroid cancer.

As shown in **Figure 1**, single-cell atlases now depict thyroid tumours as immune archipelagos in which discrete leukocyte niches co-exist and evolve along the spectrum from indolent papillary lesions to anaplastic or neuro-endocrine disease. In the 158 577-cell papillary thyroid carcinoma (PTC) compendium, T/natural-killer, B-cell, myeloid, endothelial and fibroblast lineages were resolved across primary, nodal and radio-iodine–refractory sites, exposing a progressive skew toward immuno-suppressive states as tumours dedifferentiate (38). Complementary integration of 24–202 cells from anaplastic thyroid cancer (ATC) extended this continuum, revealing an immune landscape dominated by checkpoint-positive T-cell exhaustion and polarised M2 macrophages that far outweighed cytotoxic or NK compartments (39).

Within the T-cell compartment, single-cell trajectories chart a stepwise erosion of effector capacity. Elderly tumours enrich CD28-null TEMRA cells and M2c macrophages, arguing for dose-modified ICB ± IL-7. Early-stage PTC harbours FOXP3^+^ regulatory CD4^+^ subsets and PD-1hi TIM-3^+^ exhausted CD8^+^ clusters that expand in nodal and distant lesions, while intermediate GZMK^+^ “pre-exhausted” cells fade, mirroring diminished granzyme and cytokine signatures (40, 41). ATC accentuates this pattern: LAG-3, HAVCR2 and TIGIT co-expression defines a terminally dysfunctional CD8/γδ T-cell pool whose clonal diversity contracts in parallel with rising tumour mutational burden, suggesting neo-antigenic pressure but failed immune pruning (42, 43). Such hierarchies nominate exhaustion intermediates as potential rescue points for combinatorial PD-1/LAG-3 blockade.

Myeloid lineages likewise remodel with dedifferentiation. In PTC, CCL18^+^ SPP1^+^ macrophages congregate at invasive fronts and lymph-node metastases, engaging malignant thyrocytes through SPP1–CD44 and GAS6–AXL axes to reinforce epithelial–mesenchymal transition and angiogenic signalling (44, 45). ATC intensifies the shift toward M2 phenotypes while depleting IL-12–competent M1 fractions; concurrent down-regulation of antigen-presentation genes (HLA-DRA, CD74) and up-regulation of scavenger receptors endows these macrophages with powerful T-cell suppressive capacity (20, 22). These insights rationalise macrophage-reprogramming approaches—e.g., CD47 blockade or CSF-1R inhibition—as partners for checkpoint therapy in aggressive disease.

Contrasting this immune paralysis, indolent PTCs showcase a surprisingly vibrant humoral niche. Single-cell dissection of 74–714 cells from early microcarcinomas identified enrichment of germinal-centre B cells and plasma-cell clusters that assemble tertiary lymphoid structures and correlate with favourable doubling times and disease-free survival (46, 47). Tumour cells in these cases secrete PTPRC-binding ligands that sustain B-cell proliferation, and bulk-TCGA analyses confirm higher B/plasma-cell scores in low-risk stages, suggesting that humoral immunity may act as a natural brake on progression and could be leveraged as a biomarker for active-surveillance algorithms.

Distinct immunological idiosyncrasies emerge in medullary thyroid cancer (MTC). Adrenal cancers are neutrophil-hot whereas parathyroid lesions are VISTA-macrophage-dominated, underscoring endocrine divergence. A 228 400-cell atlas comparing MTC with PTC uncovered a globally “cold” micro-environment, yet pinpointed calcitonin gene-related peptide (CGRP) secretion by para-follicular tumour cells as a deterministic cue: CGRP impedes dendritic-cell maturation via cAMP–KLF2 signalling, crippling antigen presentation and propagating T-cell dysfunction. Small-molecule CGRP-receptor antagonists (e.g., rimegepant, telcagepant) or anti-CGRP antibodies rescue CD86^hi^ HLA-DR^hi^ dendritic cells and reinstate CD8^+^ priming, underscoring translational trial opportunities (48, 49). These atlases converge on a unifying principle: thyroid cancers comprise juxtaposed “hot”, “cold” and “excluded” immune microniches whose prevalence is dictated by tumour lineage state, anatomical site and neuro-endocrine cross-talk. Mapping these niches at single-cell resolution not only illuminates mechanisms of response and resistance to checkpoint blockade, but also unveils subtype-specific liabilities—from TLS-promoting B-cell axes in indolent PTC, through macrophage-driven immune suppression in ATC, to neurotransmitter-mediated DC paralysis in MTC—that can be exploited for personalised immunotherapeutic design.

## 4 Translating single-cell insights into personalised immunotherapy strategies

Single-cell atlases have moved thyroid-cancer immunotherapy from empirical drug testing toward a data-driven, patient-in-context paradigm. By resolving “hot”, “cold” and “excluded” niches in the same tumour section, they enable dynamic rather than static triage of candidates for immune-checkpoint blockade (ICB) and allied modalities.Stratifying who benefits from ICB. The earliest proof-of-principle comes from indolent papillary micro-carcinoma, where single-cell transcriptomics revealed an expanded germinal-centre B-cell population that organises tertiary lymphoid structures and restrains tumour doubling time. Patients with this B-cell–rich signature show higher PD-L1 transcripts yet lower TIDE scores, predicting stronger responses to anti-PD-1/PD-L1 antibodies (50). Conversely, an anaplastic-thyroid-cancer (ATC) compendium uncovered pervasive CD8^+^-T-cell exhaustion (PD-1/LAG-3/TIM-3 co-expression) and M2-skewed macrophages, nominating dual PD-1^+^ LAG-3 blockade or macrophage-reprogramming as rational combinations for these “cold” tumours (51).

Single-cell ligand-receptor mapping has exposed actionable macrophage–tumour crosstalk axes. An SPP1^+^/VSIG4^+^ tumour-associated-macrophage programme, transcriptionally linked to angiogenic VEGFA and epithelial–mesenchymal transition, coincides with radio-iodine refractoriness and predicts failure of PD-1 monotherapy. Pharmacological or genetic VSIG4 ablation restored CD8-T-cell cytotoxicity and curtailed metastatic spread in murine models (52). Complementary datasets identified dendritic-cell suppression mediated by an Annexin-A1–FPR1 circuit; inhibiting this axis reinvigorated antigen presentation and synergised with anti-PD-1 in ex-vivo slice cultures (53). Such findings argue for routine myeloid-state profiling—via scRNA-seq or targeted Nanostring panels—before escalating to macrophage or DC-targeted adjuncts (e.g., CSF-1R, SIGLEC15 or FPR1 antibodies). Chronic TSH suppression dampens HLA via THRB-NRF1, potentially blunting ICB efficacy.

Heterogeneous antigen loss has hampered chimeric-antigen-receptor (CAR) strategies in solid cancers, but single-cell inventories of surfaceomes are beginning to de-risk targets in thyroid malignancy. Integrating ten scRNA-seq datasets with normal-tissue atlases pinpointed thyrotropin receptor (TSHR) as broadly retained across differentiated and metastatic lesions while largely absent from vital organs. Pre-clinical TSHR-CAR-T cells eliminated orthotopic xenografts, and optimisation work streams aimed at enhancing persistence have recently been reported (54). Parallel efforts are exploring CLDN10- and B7-H3-directed CAR constructs informed by malignant-cell clusters enriched for these epitopes. Relapse biopsies show TSHR loss and TGF-β stroma; HDAC or heparanase co-therapy may avert escape.

Visium and Slide-seq overlays show that PD-L1-high tumour islands frequently abut VEGFA-rich peri-necrotic belts, while CXCL13^+^ tertiary lymphoid structures reside millimetres away yet harbour distinct cytokine profiles. Such maps advocate centimetre-scale intratumoural heterogeneity sampling before therapy and justify image-guided intralesional injections—e.g., oncolytic viruses or Toll-like-receptor agonists—into “cold” sectors to convert them into ICB-responsive zones (55). Single-cell read-outs now underpin organoid and tumour-slice co-cultures that pair patient T-cell clonotypes with autologous tumour cells. Drug-perturbation coupled to high-throughput CITE-seq quantifies real-time checkpoint modulation, enabling rapid testing of kinase-plus-ICB cocktails under the very micro-environmental constraints catalogued *in vivo*. Early feasibility studies have demonstrated concordance between ex-vivo response and clinical outcome in advanced papillary and anaplastic cohorts, setting the stage for adaptive trial designs in which single-cell pharmacotypes dictate arm allocation. These translational advances endorse a three-step workflow: (1) upfront single-cell/spatial profiling from fine-needle aspirates or surgical specimens; (2) algorithmic assignment to immune archetypes that nominate ICB alone, ICB + myeloid modulators, or antigen-directed cellular products; and (3) real-time refinement through ex-vivo pharmacotyping and longitudinal liquid-biopsy monitoring.

## 5 Challenges and future directions

The past five years have converted thyroid-cancer atlases from blue-sky academia into pilot biomarker platforms, yet decisive clinical adoption still confronts several practical bottlenecks. Fresh-tissue dependence remains the foremost hurdle: Fine-needle aspirates (FNAs), though minimally invasive, often yield <1–000 cells, forcing nuclei-based chemistries that under-represent membrane proteins such as TSHR and B7-H3 and deplete fragile granulocytes and stromal fractions, thereby skewing immune estimates and compromising classifiers. Ambient RNA from ruptured colloid follicles can inflate epithelial transcripts, so decontamination tools such as SoupX are essential to avoid spurious PD-L1 calls. Clinical rollout demands CLIA validation plus FDA IDE/PMA clearance for single-cell diagnostic software. Harmonised protocols for cold-chain transport, nuclei-based chemistries, and preservative-enhanced dissociation are therefore required before single-cell read-outs can be embedded into routine cytopathology workflows.

Even when cellular yield is adequate, cross-platform harmonisation and computational reproducibility lag behind discovery output. Divergent alignment pipelines, batch-effect corrections, and cell-state taxonomies can re-assign >30% of malignant clusters when the same raw data are processed by different groups, undermining biomarker portability. Doublet artefacts are common in densely collagenised anaplastic tumours; cell-hashing or Freemuxlet pipelines should be deployed for reliable singlet recovery. Benchmarking consortia analogous to The Cancer Genome Atlas but centred on raw single-cell signal, reference annotations, and version-locked analysis notebooks will be critical. Machine learning toolkits that marry variational auto-encoders with graph convolution now promise near-real-time integration of transcriptome, chromatin, and protein layers, yet require prospective validation in blinded clinical cohorts.

Spatial transcriptomics begins to redress sampling bias by tethering dissociated phenotypes back to their histological coordinates, but current capture areas (≈ 55 µm spots on 10x Visium or 10 µm beads on Slide-seq v2) are too coarse to fully resolve endothelial–immune synapses or follicular-cell rosettes that dictate checkpoint engagement. Moreover, capital expenditure, sequencing depth, and analytic complexity limit deployment to specialist centres. New chemistry iterations that couple *in-situ* ligation with rolling-circle amplification could democratise sub-cellular mapping, while federated learning frameworks may allow cross-institutional transfer without raw-data sharing. Bridging descriptive atlases to actionable therapies further demands functional surrogates that preserve autologous immune and stromal constituents. Patient-derived organoids from papillary and anaplastic tumours have entered phase-II feasibility studies as drug-testing avatars, yet they currently under-represent myeloid and endothelial compartments and require week-long expansion timelines incompatible with aggressive disease courses. Air-liquid interface cultures, micro-fluidic tumour-slice arrays, and hybrid spheroid-cytotoxic-T-cell co-cultures aim to compress assay duration to 96 hours while retaining immunogenic fidelity. Regulatory clarity on how such ex-vivo pharmacotypes dovetail with Investigational Device Exemption (IDE) pathways will be essential before they can guide trial randomisation.

On the target-discovery front, single-cell atlases have spotlighted immunosuppressive myeloid niches—most notably IL2RA^+^VSIG4^+^ macrophages and SIGLEC15-enriched malignant thyrocytes—as recurrent barriers to checkpoint efficacy in anaplastic disease. Dedicated monoclonal antibodies, small-molecule degraders, and bispecific formats are advancing in pre-clinical pipelines, but careful orchestration with kinase inhibitors and anti-angiogenics will be required to avoid redundant toxicities and metabolic exhaustion of effector lymphocytes.

Equitable implementation hinges on cost and data-sovereignty considerations. Cloud-based pipelines reduce compute overhead but raise privacy concerns, particularly for geographically tagged rare-tumour datasets. Differential-privacy algorithms and on-device inference, combined with multi-modal federated learning, offer potential solutions, yet demand concerted policy engagement from regulators and patient-advocacy groups. the coming decade must convert single-cell and spatial technologies from boutique explorations into certified clinical companions. Success will rest on standardised sampling, interoperable analytics, rapid functional assays, and disciplined, combination-therapy trials that explicitly target the immune-evasive niches these atlases expose.
